# Master of Public Health in Health Equity and Criminal Justice: Student and Alumni Feedback on the Development of a New Master of Public Health Concentration

**DOI:** 10.1089/heq.2021.0055

**Published:** 2022-02-25

**Authors:** Matthew Green, Alexandra L. Hernandez, Nemesia Kelly, Carly Strouse, Trina Mackie, Gayle Cummings, Elena O. Lingas

**Affiliations:** Public Health Program, College of Education and Health Sciences, Touro University California, Vallejo, California, USA.

**Keywords:** master in public health, mass incarceration, curriculum concentration, health equity, MPH concentration, criminal justice

## Abstract

**Purpose:** To describe Master of Public Health (MPH) student and alumni interest in a new Health Equity and Criminal Justice (HECJ) concentration, highlight their personal experiences with mass incarceration, and summarize their input on developing the concentration.

**Methods:** From July to October 2017 current MPH students and alumni at Touro University California (Vallejo, CA) were electronically surveyed.

**Results:** The 152 respondents included those who had focused exclusively on public health, and those who concurrently obtained clinical degrees in osteopathic medicine, pharmacy, or physician assistant studies. Approximately 90% of the current and former students surveyed believed HECJ to be an integral part of public health, and one in three respondents described being personally impacted by incarceration. More than half (64%) were interested in the HECJ concentration, and 81% of those respondents were interested in completing their field study internship at a correctional facility.

**Conclusion:** The HECJ concentration will fill an educational gap and may provide a pedagogical model for training a future generation of public health professionals to mitigate the health impacts of the U.S. mass incarceration epidemic.

## Introduction

The United States detains more people per capita than any other nation in the world, with five times the incarceration rate of most industrialized nations despite similar crime rates.^1^ More than two million people are currently confined in federal, state, and military prisons, local jails, juvenile correctional facilities, and immigration detention facilities, reflecting an increase of 500% over the past four decades.^1,2^ The impacts of incarceration also persist after release: approximately five million people in the United States are under direct supervision through probation or parole services.^3^

Pervasive institutional racism plagues the U.S. criminal justice system and places disproportionate health, psychological, social, and economic burdens on communities of color, who are 67% of the total incarcerated population, while accounting for only 37% of the U.S. population overall.^1^ One in three black men in the United States can expect to be imprisoned at least once in their lifetime, and despite similar rates of crime perpetration, black men are more than five times as likely to be incarcerated as white men, and Latino men three times as likely as white men.^4^ These racial disparities in incarceration rates persist for women, who in 2018 made up eight percent of the total incarcerated population.^1^

Being incarcerated presents unique health concerns for those incarcerated, such as increasing the risk of infectious disease transmission.^5–8^ In addition, as individuals in the United States often serve long sentences, >40% of incarcerated individuals are living with at least one chronic condition, including hypertension, asthma and diabetes.^9^ Compared with the general population, the risk of mental illness is two to four times higher among incarcerated individuals.^10,11^ Fifteen percent of men and 31% of women in jails have a serious mental illness such as schizophrenia, major depression, and bipolar disorder.^12^ Substance abuse is also prevalent among incarcerated individuals: 68% of jailed individuals and over half of all individuals in state prisons have a diagnosed substance use disorder, five to seven times higher than the general population rates.^13^

The relationship between mental health and incarceration is dynamic: mental illness can place individuals at higher risk of incarceration, whereas being incarcerated can also further exacerbate these issues. Furthermore, although incarcerated individuals face all these health issues from incarceration, their access to health services while incarcerated may be limited, or in fact put their health at risk. The California Department of Corrections and Rehabilitation (CDCR) has been sued over the inadequate health services provided for incarcerated individuals, and CDCR has been under federal receivership since 2006.^14^

Upon release, the formerly incarcerated also lack meaningful access to health care and basic needs. Individuals are often released without necessary medications or plans for follow-up health care.^15^ They disproportionately use emergency departments for primary care purposes and have higher rates of preventable hospital admissions compared with the general population.^16^ Many re-enter society with no housing, employment, or social support, and may face discrimination in obtaining employment and housing. A large proportion of states still prohibit those convicted of drug felonies from accessing public assistance for housing and food.^17^ These restrictions compound the lifelong negative impacts of incarceration and inadequate access to health care services that already disproportionately burden communities of color.

The U.S. incarceration epidemic extends beyond individual-level impacts to adversely affect families and communities. It is estimated that one in three U.S. adults has been arrested by age 23,^1^ and between 70 and 100 million people in the U.S. have some type of criminal record.^18^ These records may impact numerous aspects of health and stability such as employment, housing, public assistance, education, finances, and family.^18^ A majority of incarcerated individuals are parents, and more than five million children under 18 in the United States have had an incarcerated parent.^19^ Parental incarceration is associated with increased adverse childhood experiences, emotional difficulties, school issues, and decreased social engagement.^19^

The field of public health already intersects with the U.S. correctional system at various levels, particularly to prevent and respond to communicable disease outbreaks, to promote sanitary conditions in prisons and jails, and to assure the provision of medical and mental health care.^20^ We felt it was important to also explore the broader societal public health implications of mass incarceration in the United States and the implications of mass incarceration for issues of equity and public health. Consequently, Touro University California's (TUC) Public Health Program (PHP) began to consider developing a Master of Public Health (MPH) concentration to train students to work with communities impacted by incarceration.

As part of the concentration development process, we noted in our companion publication that community partners and potential employers were surveyed from regional and local governments, public health departments, correctional facilities, law enforcement, and other nonprofit agencies.^21^

This study surveys current and former MPH students of the TUC-PHP regarding their interests, opinions, perceived educational needs, and experience with individuals, families, and communities impacted by incarceration.

## Methods

All current and former students of the TUC-PHP program as of October 2017 were eligible to complete the survey, including students from TUC's dual degree programs: MPH/Doctor of Osteopathic Medicine (DO), MPH/Master of Science in Physician Assistant Studies (MSPAS), and MPH/Doctor of Pharmacy (PharmD). All methods were approved by the TUC Institutional Review Board before the beginning of the study, and respondents were required to give informed consent before completing the survey.

### Survey design and distribution

Cross-sectional survey consisting of demographic, yes/no, short answer, and multiple-choice questions were administered to TUC students and alumni through the data collection platform Qualtrics. The survey response period was 12 weeks from July to October 2017. All responses were anonymous.

An electronic survey link was sent to TUC's student and alumni e-mail lists. At the time of survey distribution there were 274 enrolled students, and the alumni list contained 720 contacts. The survey link was also posted to the electronic course site of several courses and also to the TUC Facebook page asking all current and former students to complete the survey. Students were offered a chance to win a $50 Amazon gift card upon completion of the survey.

The questions were designed to gauge current student and alumni interest in a new MPH concentration focused on criminal justice and public health, including curriculum topic suggestions, and respondent experience with individuals, families, and/or communities impacted by incarceration. Respondents were asked about their opinions on a number of statements regarding the U.S. criminal justice system and public health on a 5-point Likert scale with options ranging from “Strongly Disagree” to “Strongly Agree.”

### Data collection and analysis

Data were collected and managed through Qualtrics. All responses were anonymous and kept confidential, viewable only by the authors of this study.

Quantitative variables were summarized with frequency counts and proportions. The open-ended fields were analyzed by parsing out themes.

The results were first analyzed as an entire group, and then stratified by both alumni status and degree program. No *a priori* hypotheses were made regarding differences in survey responses by either current students versus alumni or among the three degree types.

### Race and gender

We asked respondents to describe their gender in an open-ended question. Respondents listing the exact phrase “Male,” “Man,” “M,” “Trans Male,” “Cis Gender Male,” were included in the “Male” category. Respondents listing the exact phrase “Female,” “Woman,” “Girl,” “cis Woman,” and “F” were included in the “Female” category. A respondent listing “Amazing” was included as “Other.”

Respondents were asked to describe their race and/or ethnicity in an open-ended question. Respondents listing the exact term “Asian,” “Korean,” “Vietnamese,” “Indian,” “Hmong,” “Filipino,” “Chinese,” were included in the “Asian” category; “African American,” “African,” or “Black” exact responses were included in the “African or African American” category; “Multiracial,” “Mixed race,” “Mixed,” “Biracial,” “Mixed-White and Native American,” “White/Mexican,” “Irish and Mexican,” and “Asian/White” exact responses were included in the “Multiracial” category; “White/European,” “White,” “Middle-Eastern,” and “Caucasian” exact responses were included in the “Caucasian” category. “Latina” and “Hispanic,” responses were included in the “Latinx/Hispanic” category; and “Other” was included as “Other.”

## Results

One hundred fifty-two current and former students responded to the anonymous survey; 109 were current students and 43 were alumni (Table 1). Most (72%) reported their gender in an open text field with responses consistent with “female.” Forty-five percent of respondents listed their race/ethnicity in an open text field with responses consistent with “Asian,” 33% as “Caucasian,” 11% as “Latinx/Hispanic,” 5% as “Multiracial,” and 3% “African or African American.” Seventy-three percent of current and former students were in the age range of 25–35 years.

**Table 1.** Touro University California Student and Alumni 2017 Respondent Characteristics

Characteristic	All participants, ***N***	Independent MPH, ***n*** (%)	MPH/MSPAS, ***n*** (%)	MPH/DO or MPH/PharmD, ***n*** (%)
Total^a^	152	32 (21)	84 (56)	33 (22)
Gender^b^				
Male	39	8 (25)	16 (19)	15 (45)
Female	109	24 (75)	68 (81)	17 (52)
Other/decline	1	0 (0)	0 (0)	1 (3)
Race/ethnicity^c^				
African or African American	4	3 (9)	1 (1)	
Asian	70	17 (53)	40 (48)	13 (39)
Multiracial	4	1 (3)	2 (2)	1 (3)
Caucasian	52	5 (16)	31 (37)	16 (48)
Latinx/Hispanic	19	6 (19)	10 (12)	3 (9)
Age				
<25	24	3 (9)	17 (20)	4 (12)
25–35	108	26 (81)	56 (67)	26 (79)
36–45	14	3 (9)	9 (11)	2 (6)
45+	2	0 (0)	1 (1)	1 (3)
Student status				
Alumni	43	18 (55)	16 (19)	9 (26)
Current student	109	15 (45)	69 (81)	25 (74)
MPH concentration				
Community health	89	17 (52)	51 (61)	21 (62)
Global health	62	16 (48)	33 (39)	13 (38)

^a^ Nonresponses account for *N* sums not totaling 152.

^b^ Respondents were asked “Please describe gender” in an open field.

^c^ Respondents were asked “Please describe your race and/or ethnicity” in an open field.

DO, Doctor of Osteopathic Medicine; MPH, Master of Public Health; MSPAS, Master of Science in Physician Assistant Studies; PharmD, Doctor of Pharmacy.

Twenty-one percent of the respondents were completing or had received an Independent MPH; 56% were MPH/MSPAS, and 22% were MPH/DO or MPH/PharmD. Fifty-nine percent of students had selected the Community Health Concentration and 41% had selected the Global Health Concentration for their MPH studies.

Most current and former students agreed (or strongly agreed) with all statements regarding public health and incarceration (Table 2). The statement receiving the highest proportion of agreed responses was “Families of incarcerated and previously incarcerated individuals are more likely to experience mental health issues such as depression” with 92% of respondents in agreement. The statement with the lowest proportion of agreed respondents was “Race/Ethnicity impacts who gets arrested, prosecuted, and incarcerated.” However, the proportion that strongly agreed was still 78%. There were no striking differences among the three MPH degree tracks on their opinions on public health and incarceration.

**Table 2.** Opinions About Public Health and Incarceration by Respondent Degree Concentration

Statement	Independent MPH (***n***=28), %	MPH/MSPAS (***n***=80), %	MPH/DO or MPH/PharmD (***n***=23), %
Families of incarcerated and previously incarcerated individuals are more likely to experience mental health issues such as depression	82	92	91
Previously incarcerated individuals have poorer health outcomes than the general population in their community	89	93	85
Economic status/class impacts who gets arrested, prosecuted, and incarcerated	89	96	85
Mass incarceration is a public health problem	86	87	85
Health equity and criminal justice are an integral part of public health	89	90	85
Immigration status impacts who gets arrested, prosecuted, and incarcerated	82	89	82
Educational status impacts who gets arrested, prosecuted, and incarcerated	86	91	82
Race/ethnicity impacts who gets arrested, prosecuted, and incarcerated	86	94	78

Respondents who “Agree” or “Strongly Agree” with the following statements regarding the United States (*N*=131).^a^

^a^ Nonresponses account for *N* not totaling 152.

A majority of respondents indicated that they would have been interested in a concentration focused on Health Equity and Criminal Justice (HECJ; Table 3). The proportion that responded favorably differed by degree track, with 50% Independent MPH, 66% of MPH/MSPAS, and 70% of MPH/DO or MPH/PharmD respondents answering affirmatively. Many of the interested respondents (*n*=85) also indicated that they would have been interested in completing a Capstone project focusing on HECJ: 77% of Independent MPH and 52% of MPH/DO or MPH/PharmD would have been interested.

**Table 3.** Interest in the Health Equity and Criminal Justice Concentration Based on Responses to Yes/No Questions

Survey question	Independent MPH (***n***=30), %	MPH/MSPAS (***n***=82), %	MPH/DO or MPH/PharmD (***n***=23), %
Respondent would have been interested in a concentration focused on HECJ if available while they were a student (*N*=135)^a^	50	66	70
Among respondents indicating they would be interested in a concentration focused on HECJ (*N*=85):			
**Characteristic**	**Independent MPH (*n*=13), %**	**MPH/MSPAS (*n*=51), %**	**MPH/DO or MPH/PharmD (*n*=21), %**
Respondent would have been interested in completing a Capstone project focusing on HECJ	77	35	52
Respondent would have liked to complete field study at a correctional facility	85	80	81
Respondent thinks it is important/useful for TUC to have this concentration in HECJ as part of the MPH program	100	100	100

^a^ Nonresponses account for *N* not totaling 152.

HECJ, Health Equity and Criminal Justice; TUC, Touro University California.

More than 80% of all interested students, regardless of degree track, reported a desire to have completed their field study at a correctional facility, and all of them responded that they believed it was important/useful for TUC to have an HECJ concentration as part of the MPH program.

Respondents who said that they would have been interested in an HECJ concentration were asked in an open text field to explain what interested them in the HECJ concentration and why they thought it would be important/useful for TUC to have this concentration as part of the MPH program. The most common responses regarding HECJ interest were consistent to the themes: “HECJ is an interesting, relevant topic,” “HECJ is integral to public health as a social determinant,” and “it would be useful to learn how to improve the situation with a public health approach.”

When elaborating on why it would be important for the TUC-PHP to have this concentration, a majority of responses were consistent with the following themes: “HECJ is integral to public health,” “there is strong student interest in HECJ, this concentration would be an additional option for students,” “HECJ is an understudied, overlooked topic,” and “HECJ resonates with TUCs commitment to social justice.”

Specific responses regarding interest in the HECJ concentration included the following:

“Health equity is a central action theme of public health, and in my opinion, the reason we do the work we do. Understanding powerful forces that impact health equity, such as criminal justice, is critical to being an effective public health leader. I would have pursued this track if available at the time.”—Current Student, MPH/DO, Community Health Concentration

“Touro is one of the only schools I've heard of that has “social justice” in its mission statement and core values dialogue. If that's true, which I believe it is for Touro, then I think it's all the more reason to make sure that subject such as this are brought to light. Difficult public health issues are what need to be worked on the most.”—Alumni, Independent MPH, Global Health Concentration

Many students also suggested including exposure or experience working directly with incarcerated individuals. For example:

“It would be very interesting to get hands on experience in learning about and witnessing the public health issues in correctional facilities.”—Current Student, MPH/MSPAS, Community Health Concentration

“The only way to learn about the conditions of a correctional facility is to witness it, rather than just reading about it.”—Current Student, MPH/MSPAS, Community Health Concentration

“I think it would be a very rare and unique experience. I think it would be emotionally heavy field study but the knowledge and insight gained from it would be priceless.”—Current Student, MPH/MSPAS, Global Health Concentration

Students and alumni who expressed interest in an HECJ concentration were asked what topics would be important to include in a curriculum for an HECJ concentration (Table 4). Of the topics listed, “Incarceration impacts on families” (91%), “Incarceration impacts on individuals” (82%) and “Incarceration impacts on communities” (82%), were highly favored over topics such as “Recidivism” and “Penalization and Crime Classification (48%).”

**Table 4.** Curriculum Development Input from Respondents Who Would Have Been Interested in the Health Equity and Criminal Justice Concentration

“What topics do you believe are especially important to cover in training students in Health Equity and Criminal Justice?”^a^	
Topic	Students and alumni (***n***=85), %
Health of incarcerated individuals	86
Incarceration impacts on families	85
Incarceration impacts on communities	79
School to prison pipeline	76
Chronic health impacts of incarceration	75
Criminology (who commits crimes and why)	67
The U.S. criminal justice system	63
Economic impacts of incarceration	62
Environmental contributors	57
Historical contributors	53
Detention systems for immigrants—past and current	51
Recidivism	48
Penalization and crime classification	48

^a^ Multiple responses possible.

Four respondents also selected “Other” and wrote in suggestions that included more narrow criminal justice topics, including “social groups formed within prison and why”; “intersection of gender identity and incarceration”; “drug policy and public health alternatives to drug criminalization”; and “undocumented immigrants.” Several included topics that were not specific to criminal justice, including “research methods in social epidemiology”; “multilevel modeling,” and “statistical programming.”

Finally, current and former student respondents were asked if they had any “personal or professional experience with individuals, families, or communities impacted by incarceration.” Thirty-five percent of students said that they did have personal or professional experience. This proportion did differ by degree track with 45% of MPH/DO or MPH/PharmD students responding with this experience, MPH/MSPAS 31% and Independent 27%. The 50 students who responded affirmatively to this question were asked to describe their personal or professional experience. Family, patients, and “work” were the most common themes reported.

## Discussion

Although public health as a field has acknowledged the public health significance of mass incarceration, the authors are not aware of any Council on Education for Public Health-accredited MPH program that offers specialized training focused on the public health implications for individuals and communities impacted by the criminal justice system. Previous study^21^ discovered that community partners and potential employers of TUC MPH graduates were strongly in favor of the development of an MPH concentration focused on the intersection of public health and incarceration. The study shows that this support is also present among current and former MPH students.

Most student and alumni respondents were in favor of the development of the HECJ concentration, and more than half would have chosen it if it had been an option at the time of their enrollment in the program. This demonstrates strong support from students for development of this concentration. More than 80% of those who expressed interest in this new concentration indicated that they would have wanted to complete their field study at a correctional facility. This is in line with findings from a previous survey of community partners and potential employers. Respondents to that survey indicated that direct experience in a correctional facility or working with formerly incarcerated individuals is one of the most important components of training in HECJ.^21^

A subset of student and alumni respondents reported previous personal or professional experience with incarcerated individuals, which affirms the scope of the issue in the United States and the number of lives it touches. They reported having experienced the impacts of incarceration through a family member, and a majority (91%) of these students would like to see “Incarceration impacts on families” covered in the curriculum. This highlights a particular focus area of interest for the development of this concentration, which will not only include the health impacts of incarceration on the individual, but also those on the family and community connected to them.

This study has a few limitations. The sample was not random because the survey link was posted to the TUC PHP Facebook page, and e-mailed to student and alumni distribution lists. These dissemination methods also made it impossible to determine if respondents were accurately reporting their affiliation with TUC. It is possible, but unlikely, that respondents falsely reported that they were students or alumni. Duplicate entries could not be prevented, but based on the variation in responses this also seems unlikely.

The respondents of this study of current and former students vary in their career paths, training, age, race/ethnicity, and concentration, yet almost all share the belief that mass incarceration is a major public health issue. This aligns with national, state, and local public health agencies.

The American Public Health Association (APHA) has long-held opposition to the social practice of mass imprisonment. APHA continues to support alternatives to incarceration and deeper evaluation of the root causes leading to the disproportionate incarceration rates of people of color^22^ and has also released a number of public health policy statements on other aspects of criminal justice: law-enforcement violence and over-policing,^23^ re-entry support,^24^ solitary confinement,^25^ mental health and substance abuse services for incarcerated individuals,^26^ smoking cessation in incarceration settings,^27^ and sex education in correctional facilities.^28^

The National Association of County and City Health Officials has also acknowledged mass incarceration as a public health crisis disproportionately impacting children, families, and communities of color.^29^ The California Department of Public Health affirms this assertion and supports mitigating community trauma by addressing issues such as community relationships with law enforcement, and mass incarceration.^30^ Furthermore, many local health departments throughout California are utilizing core public health skills to engage with the criminal justice system,^31^ and recent studies also support the assertion that mass incarceration is a public health problem.

Recent studies describe how mass incarceration drives health inequity,^32^ examine incarceration as a health risk and provide suggestions for next steps in public health research, training, and practice,^9^ and outline a call to action involving the development of new partnerships, the power of data, and an emphasis on public health-focused policy solutions.^33^ Training TUC MPH graduates in HECJ will provide them with the tools and knowledge to work effectively with individuals and communities in a variety of settings, and to respond to the research and advocacy needs to address the health inequities propagated by mass incarceration.

## Conclusions

Given the apparent student and alumni interest, the importance of the focus area for public health and population equity, and resonance with core social justice values, the PHP at TUC is moving forward with developing and implementing the HECJ concentration. This offering at TUC will be impactful due to the wide range of students trained annually, including >60 students who will be practicing as clinicians along with the MPH degree, whether as pharmacists, physician assistants, or providers of osteopathic medicine.

This is particularly relevant because there are no national standards for teaching health care workers about correctional health, and few clinical programs with related topics as part of the curriculum.^34^ The HECJ concentration will provide an opportunity to equip a new generation of clinicians and public health professionals to more deeply understand the intricacies, humanity, and context of the populations they serve.

## Abbreviations Used

APHA: American Public Health Association
CDCR: California Department of Corrections and Rehabilitation
DO: Doctor of Osteopathic Medicine
HECJ: Health Equity and Criminal Justice
MPH: Master of Public Health
MSPAS: Master of Science in Physician Assistant Studies
PharmD: Doctor of Pharmacy
PHP: Public Health Program
TUC: Touro University California
